# Evolution of pollutant biodegradation

**DOI:** 10.1007/s00253-025-13418-0

**Published:** 2025-02-04

**Authors:** Yi Ren, Mike Manefield

**Affiliations:** Water Research Centre, School of Civil and Environmental Engineering, University of New South Wales, Sydney, NSW 2052 Australia

**Keywords:** Epistasis, Promiscuity, Bacteria, Mutation, Toxicity, Resistance

## Abstract

**Abstract:**

Pollutant-derived risks to human and environmental health are exacerbated by slow natural attenuation rates, often driven by pollutant toxicity to microorganisms that can degrade them or limitations to the ability of microorganisms to metabolise them. This review explores mechanisms employed by bacteria to protect themselves from pollutant toxicity in the context of the evolution of pollutant-degrading abilities. The role of promiscuous enzymes in pollutant transformation is subsequently reviewed, highlighting the emergence of novel metabolic pathways and their transcriptional regulation in response to pollutant exposure, followed by the gene transcription regulation to optimise the cellular component synthesis for adaptation on the novel substrate. Additionally, we discuss epistatic interactions among mutations vital for this process both at macromolecular and at cellular levels. Finally, evolutionary constraints towards enhanced fitness in the context of pollutant degradation are considered, the constraints imposed by the epistasis from mutations on both enzyme level and cellular level, concluding with challenges and emerging opportunities to develop sustainable contaminated site remediation technologies.

**Key points:**

*•Pollutants can exert toxicity on cellular membrane, enzyme and gene transcription.*

*•Bacteria can patch promiscuous enzymes into novel pathway to degrade pollutants.*

*•The evolution trajectory is constrained by epistasis from mutations on enzyme and cellular level.*

## Introduction

Use of pesticides, herbicides, dyes, oil derivatives, explosives, industrial solvents, detergents and firefighting foams contributes to pollution in terrestrial and aquatic ecosystems (Geissen et al. 2015; Atashgahi et al. 2018; Rathore et al. 2022). Widespread application of these variously halogenated or nitrogenated aromatic and aliphatic compounds is a significant concern due to their resistance to biodegradation, tendency to bioaccumulate and detrimental effects on both human and environmental health (Ng et al. 2021; Ahmad et al. 2021; Wilkinson et al. 2017; Dhakal et al. 2018; Tsaboula et al. 2016; Embrandiri et al. 2016; Spahr et al. 2020; Masoner et al. 2019; Ensminger et al. 2013). The adverse impacts on human health are extensive, encompassing immune system damage, pulmonary bronchitis, nervous system dysfunction, endocrine disruption, developmental disorders, mutagenicity and carcinogenicity (Bertotto et al. 2020; Mishra et al. 2019; Catron et al. 2019; Dinka 2018; Zhu et al. 2017; Rathore et al. 2022). Pollutants are also toxic to microorganisms by adversely affecting cell membranes, DNA, ribosome function and enzyme activity (Smulek et al. 2019; Wang et al. 2020; Du et al. 2020; Fitzgerald et al. 2017). The evolution of pollutant degradation underpins natural attenuation and biological remediation applications, given legislation in many jurisdictions prohibiting release of genetically modified organisms.

Microorganisms evolve to degrade naturally occurring or synthetic pollutants as a defence against toxicity and to harvest essential elements and energy. Thus, they play a vital role in mitigating accumulation in the environment, underpinning biological remediation technologies now in widespread commercial application (Bhatt et al. 2021; Mishra et al. 2021; Varjani et al. 2017; Ashrap et al. 2017; Parmar et al. 2022; Zhan et al. 2018; Siles and Margesin 2018; Ortiz-Hernández et al. 2018). Metabolic pathways can develop by harnessing existing promiscuous enzymes, which have wide substrate ranges, albeit with initially low binding affinity and low catalytic efficiency (Copley 2000, 2017, 2020, 2021; Schulenburg and Miller 2014).

This review begins by summarising the mechanisms underlying pollutant toxicity and the corresponding bacterial detoxification strategies followed by examples of microbial adaptation in response to pollutants, highlighting the role of promiscuous enzymes in patchworking novel metabolic pathways. The subsequent optimisation of these metabolic pathways and transcriptional regulation is explored, along with analysis of epistatic interactions between mutations within enzymes and cells that constrain evolutionary trajectories (Yang et al. 2019). Finally, the review will address future research challenges and opportunities in the biodegradation of pollutants.

## Mechanisms of pollutant toxicity and resistance

Depending on their polarity, pollutants such as halogenated organics can disrupt the function of cell membranes composed of lipid bilayers (Manefield et al. 2017). For example, perfluorinated alkyl substances (PFAS) partition into the lipid bilayers of Gram-positive and -negative bacteria resulting in increased membrane permeability and proton leakage (Fitzgerald et al. 2017). PFAS can also impair membrane synthesis and function in fungi (Qiao et al. 2019).

Pollutants also generate reactive oxygen species (ROS) that interfere with redox reactions, membrane integrity, DNA/RNA integrity and enzyme activity in microorganisms (Du et al. 2020; Wielsøe et al. 2015). ROS production can stem from sub-optimal positioning of pollutant molecules when interacting with enzymes, leading to uncoupled, non-productive oxygen-delivery reactions and subsequent oxidative stress (Perez-Pantoja et al. 2013).

Enzyme activity is also directly impacted by pollutants. For example, PFAS can alter the secondary structure of catalase enzymes through interaction with aromatic amino acids (Xu et al. 2018). Chlorinated aliphatic hydrocarbons can compete for binding sites on methyltransferases (Yu and Smith 2000) and non-cognate reductive dehalogenases (Chan et al. 2010, 2011; Wei et al. 2016; Yu et al. 2005; Duchesneau et al. 2007; Wen et al. 2017).

Pollutants can also have detrimental impacts on gene expression in microorganisms, in both transcription and translation. For example, halogenated hydrocarbons like chloromethane act as alkylating agents introducing methyl groups to RNA nucleobases and ribose. Such alkyl adduction disrupts base pairing geometry in RNA polymerases and ribosomes (Yan and Zaher 2019).

Microorganisms employ various mechanisms to alleviate the toxicity of pollutants including expulsion, conjugation or degradation. Transmembrane transporters like the ABC transporter family, particularly those with relaxed ligand specificity, defend against pollutant toxicity either through expulsion or by importing pollutants for conjugation or degradation (Ecker et al. 2008; Teichmann et al. 2017; Desai and Miller 2010; Banerjee et al. 2000). Conjugation with glutathione via glutathione transferase is a well-known means of altering the polarity of a pollutant thereby reducing toxicity or enabling expulsion (Shoji 2014). Broad spectrum peroxidases also methylate pollutants reducing toxicity (Badkoubi et al. 1996). In all cases, enzyme promiscuity enables some level of resistance whether pollutants are naturally occurring or xenobiotic. There are also a multitude of ways microorganisms can degrade pollutants including non-specific and specific oxidation and reduction reactions. The remainder of the review focusses on the evolution of such traits that can be exploited in sustainable remediation technologies.

## Degradative enzyme multiplicity

Microorganisms exploit multiple copies of genes encoding degradative enzymes, enabling mutation without loss of viability because of functional redundancy. As an example, organohalide respiring bacteria harbor multiple reductive dehalogenase genes that confer an ability to dechlorinate and hence reduce the toxicity of these pollutants (Jugder et al. 2015; Rupakula et al. 2013). The gene multiplicity enables them to accumulate mutations that can expand the substrate range without compromising the ability to harvest energy from the dechlorination reaction (Tokuriki and Tawfik 2009; Khersonsky et al. 2006). Interestingly, error-prone DNA repair mechanisms can foster the evolution of pollutant-degrading strains by attacking the C-8 position of guanine in deoxyguanosine and guanosine, causing DNA and RNA damage and enhancing mutagenesis (Du et al. 2020), providing a potential avenue for adaptive responses to environmental stressors (Akkaya et al. 2018; Wielsøe et al. 2015; Cooke et al. 2003).

*Dehalococcoides ethenogenes* strain 195 harbors 19 dehalogenase genes including *pceA* encoding tetrachloroethene dechlorination. The tetrachloroethene dehalogenase also accepts 2,3-dichlorophenol as a substrate, suggesting it evolved from an enzyme targeting naturally occurring chlorophenols on exposure to the xenobiotic solvent (Fung et al. 2007). *Dehalobacter restrictus* strain UNSWDHB, isolated in Australia from chloroform and 1,1-dichloroethane contaminated groundwater, encodes 17 full-length and 3 truncated reductive dehalogenase genes (Wong et al. 2016; Lee et al. 2012). One of these dehalogenase genes is *tmrA*, encoding a chloroform reductase which also accepts 1,1-dichloroethane as substrate (Lee et al. 2012). Interestingly, a closely related strain isolated in Canada from 1,1,1-trichloroethane contaminated groundwater encodes a very similar chloroform reductase (*cfrA*) which has strong activity against 1,1,1-trichloroethane but low activity against 1,1-dichloroethane (Picott et al. 2022). Dehalogenase gene multiplicity appears to have enabled evolution in response to the pollutant profile within decades delimited by the contamination event and the isolation of the bacteria.

## Novel biodegradation pathways assembled from promiscuous enzymes

Novel metabolic pathways for pollutant degradation evolve by leveraging existing promiscuous enzymes and exploiting structural similarities between the pollutants and natural enzyme substrates. Transformation intermediates can then be further metabolised by other existing enzymes, thereby forming novel metabolic pathways (Shoji 2014; Wackett 2004). This is exemplified by the evolution of novel biodegradation pathways for anthropogenic aromatic pollutants with chloro or nitro groups which resemble naturally occurring aromatics used as carbon and energy sources (Ju and Parales 2009; Liu et al. 2011).

**Fig. 1:**
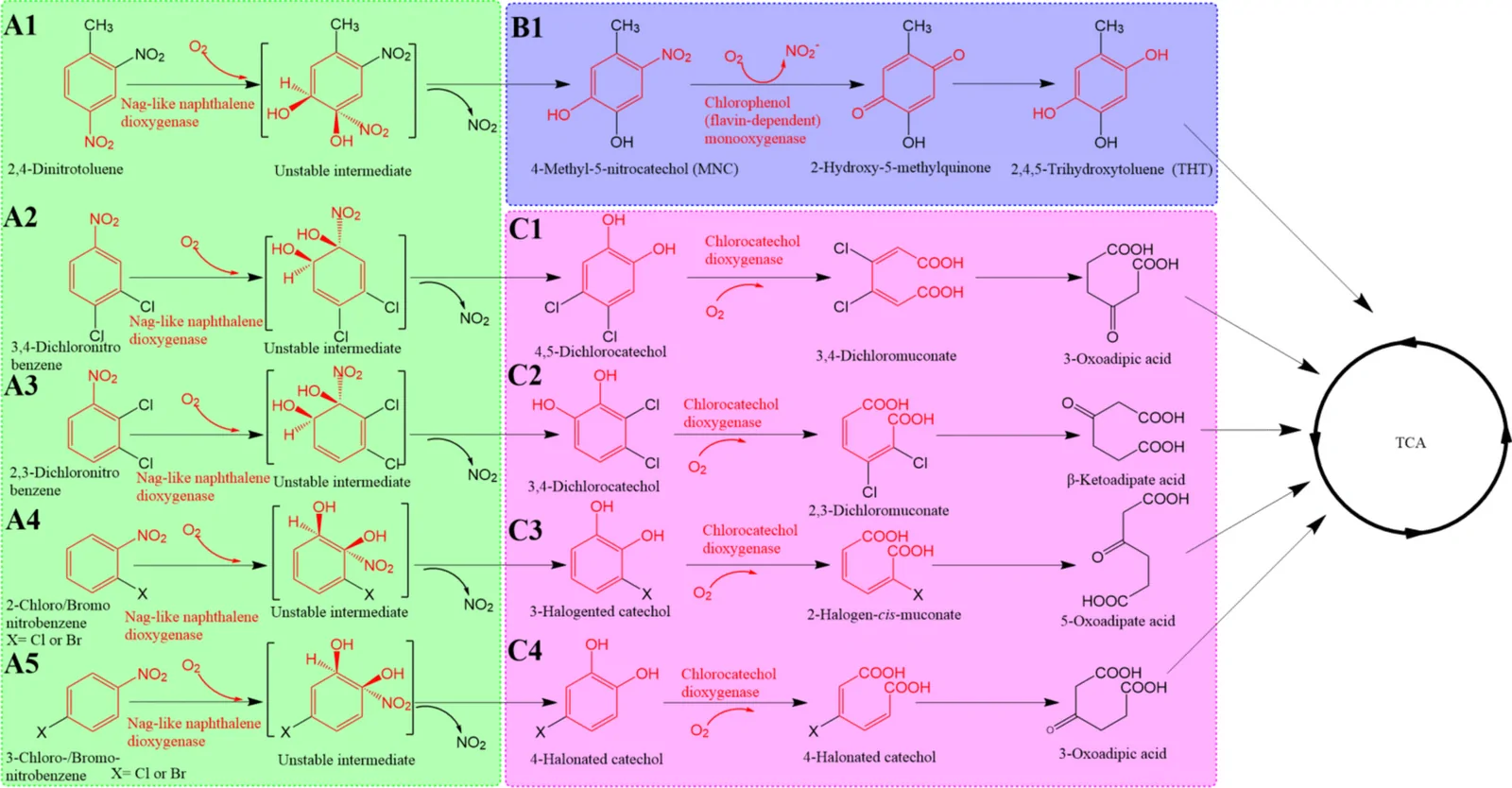
Both upstream and downstream novel metabolic pathways were developed by incorporating promiscuous enzymes.The upstream reactions (A1–A5), depicted in green, are catalysed by Nag-like naphthalene dioxygenase, with red highlighting common chemical structures and reactions recognised by this enzyme. The downstream reactions are shown in blue and pink (B1 and C1–C4). Reaction B1 is catalysed by a recruited FMO, with red indicating common chemical groups and reactions recognised by the enzyme. Reactions C1–C4, catalysed by a recruited chloro-catechol dioxygenase, also featured in red to denote common chemical structures and reactions recognised by this enzyme

The microbial degradation of halo/nitroaromatic pollutants encompasses two phases (Fig. 1). The upstream phase involves Nag-like naphthalene dioxygenases to oxidise these pollutants into halo/nitro catechols (Ju and Parales 2009; Liu et al. 2011) (Fig. 1, A1–A5). The downstream phase proceeds via two pathways for processing of halo/nitro catechols. The first pathway involves flavin-dependent monooxygenases (Pimviriyakul et al. 2018; Pimviriyakul and Chaiyen 2018) that hydroxylate halo/nitro catechols into hydroquinone derivatives (Fig. 1, B1). The second pathway utilises catechol dioxygenases that catalyse *ortho-ring* cleavage generating adipic acid derivatives (Fig. 1, C1–C4). These enzymatic transformations facilitate the integration of pollutant degradation products into the tricarboxylic acid (TCA) cycle, thus supporting microbial growth and survival (Ju and Parales 2009).

Nag-like naphthalene dioxygenases feature a large substrate binding pocket and flexible loops at the entrance channel, enabling them to accommodate a wide range of aromatic substrates (Jouanneau et al. 2006; Khara et al. 2018). For example, *Acidovorax* sp. strain JS42 catalyses 4-nitrotoluene (4-NT) transformation with an adapted Nag-naphthalene dioxygenase (Ju and Parales 2011). This adaptation involves only three missense mutations to broaden the substrate range. Such adaptation can also be found in Nag-naphthalene dioxygenases from *Burkholderia cepacia* strain R34 for 2,4-dinitrotoluene degradation (Fig. 1, A1), *Diaphorobacter* sp. strain JS3050 for 3,4-dichloronitrobenzene degradation (Fig. 1, A2), *Pseudomonas stutzeri* strain ZWLR2-1 for 2-chloro/bromo-nitrobenezene degradation (Fig. 1, A4) and *Diaphorobacter* sp. strain JS3051 for 2,3-dichloronitrobenzene and 3-chloro/bromo-nitrobenzene degradation (Fig. 1, A3 and A5) (Gao et al. 2021; Li et al. 2021; Liu et al. 2011; Wang et al. 2019).

Flavin-dependent monooxygenases (FMOs) are enzymes with great redox versatility capable of hydroxylation of aromatic or aliphatic hydrocarbons (Sobrado 2012). They are widely distributed among soil bacteria, because phenolic compounds derived from lignin represent an important carbon source (Copley et al. 2012). FMOs catalyse similar hydroxylation reactions on a range of pollutants with analogous structural features, especially the substituent group at the *para* position to the hydroxyl group. In the case of aromatic pollutants, FMOs target molecules with halide or nitro groups substituted at the *para* position (Pimviriyakul et al. 2018) which are eliminated to form benzoquinone (Pimviriyakul et al. 2021).

For example, in the degradation pathway of 2,4-dinitrotoluene in *Burkholderia cepacia* strain R34, a Nag-like naphthalene dioxygenase produces 4-methyl-5-nitrocatechol (MNC) (Fig. 1, B1), which is oxidised to 2-hydroxy-5-methylquinone by MNC monooxygenase via the same hydroxylation reaction mechanism of FMOs. In *Ralstonia pickettii* DTP0602, the FMO HadA catalyses oxidative dehalogenation and denitration of chlorophenols and nitrophenols due to their structural resemblance. By introducing hydroxyl groups to the *para* positions, HadA converts chlorophenols and nitrophenols into hydroquinones, which are integrated into the TCA cycle (Pimviriyakul and Chaiyen 2018; Pimviriyakul et al. 2018, 2017). The promiscuous activity of HadA towards substrates with *para* substituents is linked to specific conserved residues within its active site. Histidine 290 is crucial for facilitating the dehalogenation and denitration of phenols (Pimviriyakul et al. 2021). Additionally, the presence of two phenylalanine residues at the active site forms an ‘aromatic cage’ that positions the *para* position of the aromatic ring in close proximity to the flavin cofactor (Pimviriyakul et al. 2018, 2021). This allows HadA to modify halogen or nitrogen groups at the *para* position, underscoring its ability to degrade environmental pollutants (Pimviriyakul et al. 2018). The FMOs phenol 4-monooxygenase from *Arthrobacter chlorophenolicus* strain A6, 4-fluorophenol (4-FP) monooxygenase from *Arthrobacter* sp. strain IF1 and PnpA1 from *Rhodococcus imtechensis* strain RKJ300 hydroxylate *para* substituted chlorophenols, fluorophenols and nitrophenols, respectively (Cho et al. 2017; Ferreira et al. 2009, 2008; Guo et al. 2021). Beyond single halogenated phenols, the multi-halogenated pesticide pentachlorophenol (PCP) is degraded into tetrachlorobenzoquinone by the enzyme PcpB from *Sphingobium chlorophenolicum* L-1 (Copley et al. 2012; Copley 2000). The presence of a flavin cofactor in PcpB aligns it with the FMO family, highlighting the adaptability of FMO enzymes to target a wide array of aromatic pollutants (Copley et al. 2012; Reis et al. 2021; Sobrado 2021).

Catechol dioxygenases are another promiscuous group of enzymes for downstream halo/nitro aromatic pollutant degradation, acting through intradiol-cleavage of the aromatic ring (Christian et al. 2016; Zeng et al. 2020; Costas et al. 2004). The crystal structure of chlorocatechol dioxygenase reveals heterogeneity in the conformation of co-crystallised ligands indicative of structural plasticity within the cavernous active site enabling broad substrate range (Ferraroni et al. 2006; Zhang et al. 2022). For example, catechol dioxygenase Tcu3516 from *Thermomonospora curvata* strain DSM43183 exhibits activity not only for 3-chlorocatechol and 4-chlorocatechol (Fig. 1, C3 and C4) but also for larger substrates such as 2,3-dihydroxybiphenyl and 3,4-dihydroxyphenylacetic acid (Zhang et al. 2022). This promiscuity is crucial for integrating catechol dioxygenase into downstream pathways for the degradation of halo/nitroaromatic pollutants. *Diaphorobacter* sp. strain JS3051 utilises the catechol-1,2-dioxygenase to complete the downstream degradation pathways of 4,5-dichlorocatechol and 3,4-dichlorocatechol (Fig. 1, C1 and C2) (Gao et al. 2021; Li et al. 2021) and in degrading 4-chloro/bromo-catechol (Fig. 1, C4), dovetailing with the upper pathway degradation of 3-chloro/bromonitrobenzenes (Xu et al. 2022). *Pseudomonas stutzeri* strain ZWLR2-1 converts 3-chloro/bromo-catechol into 5-oxoadipic acid using catechol-1,2-dioxygenase. This enzymatic process is integral to completing degradation of 2-chloronitrobenzene (2-CNB) and 2-bromonitrobenzene (2-BNB) (Liu et al. 2011; Wang et al. 2019).

In summary, a variety of pre-existing enzymes with promiscuous activities can be co-opted for pollutant degradation. Substrate promiscuity is largely due to spacious active sites, hydrophobic residues that contribute to higher binding affinity with aromatic pollutants (Jouanneau et al. 2006; Khara et al. 2018), and specific conserved residues in the active sites that carry out versatile reactions across different substrates (Pimviriyakul et al. 2021). Additionally, the versatility of cofactors, with varied redox potentials, broadens the spectrum of possible redox reactions. To integrate pollutant degradation into central metabolism, novel metabolic pathways emerge through promiscuous enzymes acting in series.

Taken together, microorganisms harbour enzymes with promiscuous activities that can be applied for bioremediation. For the degradation of recalcitrant organohalogens, *Dehalobacter*-containing mixed cultures, including those with chloroform reductase (cfrA), have been shown to dechlorinate 1,1,1-trichloroethane in contaminated groundwater in Canada (Picott et al. 2022). Similarly, *Dehalobacter* UNSWDHB, from a chloroform-contaminated site in Australia, can degrade chloroform into dichloromethane (Wong et al. 2016), with Dehalobacter-rich cultures further metabolising chloroform into acetate and hydrogen without accumulating toxic intermediates (Lee et al. 2012). For persistent halo/nitroaromatic pollutants, engineered bacterial strains leveraging promiscuous nitroarene dioxygenases can mineralise pesticides like chloronitrobenzene into central metabolic pathways (Ju and Parales 2009). Furthermore, understanding the atomic-level details of enzymes such as HadA monooxygenase, which exhibit promiscuous activity against halo/nitroaromatic pollutants, offers valuable insights. These can guide enzyme modifications to enhance dehalogenation and denitration efficiency, thereby improving bioremediation processes (Pimviriyakul and Chaiyen 2018).

## Optimisation of gene transcription regulation

While microorganisms can derive energy from promiscuous reactions with pollutants, transcriptional regulation in these scenarios may not be optimal. The main challenge is the potential disconnect between transcriptional regulation and the presence of the pollutant because transcriptional regulators may not be as promiscuous as the regulated enzyme and therefore do not recognise the pollutant as substrate. Consequently, mutations optimising transcription can be selected for through enhanced energy or element harvesting from the pollutant or through detoxification.

Guzman et al. (2019) demonstrate the adaptive capabilities of *Escherichia coli* strain MG1655 in optimising gene expression for d-arabinose utilisation using laboratory evolution experiments. The initial adaptation to metabolise d-arabinose was sub-optimal, yielding less energy compared to native substrates. A mutation in the transcriptional regulator *araC* increased d-arabinose binding affinity resulting in upregulation of genes encoding arabinose metabolism (Schleif 2010; Utrilla et al. 2016).

Similarly, *Rhodococcus opacus* strain PD630 evolved enhanced phenol utilisation in another laboratory evolution experiment, primarily through the optimisation of gene expression (Yoneda et al. 2016). Transcription data revealed that adapted strains upregulate a second copy of a two-component phenol hydroxylase gene, the downstream catechol 1,2-dioxygenase gene and other downstream genes in the beta-ketoadipate pathway. Additionally, a mutation in an upregulated transporter gene increased the intracellular phenol concentration thus activating the transcriptional regulator.

The transcriptional regulation of enzyme expression with promiscuous activities can also become constitutive or upregulated in response to alternative inducers. Copley (2000) observed that the maleylacetoacetate (MMA) isomerase of *Sphingomonas chlorophenolica* evolved a novel activity for the reductive dehalogenation of tetrachlorohydroquinone. Following this adaptation, the transcription of MMA isomerase became constitutive, allowing the enzyme to be expressed under conditions where its native activity is not required. This shift to constitutive expression enables the enzyme to function effectively in a broader range of environmental contexts and respond more readily to novel substrates.

In *Pseudomonas* sp. strain ADP, a genetic adaptation involved the acquisition of *atzA* and *atzB* genes, which are crucial for the degradation of the herbicide atrazine. Interestingly, the expression of these genes has been observed even in the absence of atrazine or hydroxyatrazine, which are typically required as inducers. Bioinformatics analysis did not identify any regulatory elements upstream of these two genes, indicating that *atzA* and *atzB* are constitutively expressed in this strain (Martinez et al. 2001; Govantes et al. 2010). Such constitutive expression suggests an evolutionary trade-off, allowing the bacteria to degrade atrazine whenever it is encountered in the environment, without the need for prior exposure to the herbicide or its derivatives to trigger gene activation. This adaptability in gene expression enhances the capacity to respond promptly to environmental pollutants like atrazine, though there is a trade-off requiring more cellular resources to be invested in times when the pollutant is not present (Goelzer and Fromion 2017).

*Acidovorax* sp. strain JS42 shows regulatory adaptation for degradation of the carcinogenic chemical manufacturing precursor 2-nitrotoluene (2-NT) degradation (Mulla et al. 2013). This adaptation involved utilising genes originally for naphthalene degradation. The key to this process is the adaptation of the transcription regulator NtdR, which only differs by five amino acids from the original naphthalene degradation regulator, NagR. While both NtdR and NagR can activate gene expression in the presence of salicylate (a naphthalene degradation intermediate and natural inducer for naphthalene degradation genes), NtdR exhibits a broader substrate range (Ju et al. 2009). This expanded inducer specificity of NtdR includes not only 2-NT but other nitroaromatic compounds enabling bacteria to respond to a wider array of environmental pollutants by expressing relevant degradation pathways.

## Epistasis in fitness landscapes

Evolution of a pollutant degrading organism from a non-degrader would typically involve multiple mutations occurring one after the other to ultimately achieve superior evolutionary fitness. The benefits of specific mutations on fitness are contingent on the pre-existing genetic background, such that initial mutations towards pollutant degradation may decrease fitness with subsequent mutations achieving higher fitness (Szappanos et al. 2016; Chou et al. 2014). This dependency of fitness changes induced by new mutations on the pre-existing genetic background is known as epistasis. Epistatic effects can be observed within an enzyme (intramolecular) or within a cell (intermolecular). Interactions between sequential mutations determine the probability of new pollutant-degrading abilities emerging (Weinreich et al. 2005, 2006; Poelwijk et al. 2011). The evolutionary trajectory toward higher fitness has been conceptualised to occur in a ‘fitness landscape’, in which there are myriad possible sequences of mutations to optimised fitness, with troughs of low fitness potentially required to be overcome to reach new fitness peaks (de Visser and Krug 2014; Poelwijk et al. 2007). The probability of certain amino acid residues changing can be calculated based on entropy (Voigt et al. 2001). The number of amino acid substitutions that can be made at a given residue position without compromising structural integrity has revealed a strong correlation between the probability of beneficial mutations and high-entropy positions (Mullick et al. 2021).

As an example of intramolecular epistasis in enzyme evolution, Wang et al. (2002) and Weinreich et al. (2006) investigated emergence of cefotaxime hydrolysis by β-lactamase. They identified the five key point mutations required, but realised the order in which these mutations occurred was crucial. Of the 120 possible permutations, only 18 pathways across the fitness landscape were viable due to epistatic constraints. Specific mutations enhanced enzyme activity against cefotaxime but compromised the thermal stability of the enzyme. For example, fixing a noncoding mutation (g4205a) that increased gene transcription but led to protein aggregation (reduced thermal stability) was contingent on another mutation (M182T) that enhanced thermal stability occurring beforehand. Noor et al. (2012) investigated the evolutionary transition from TriA (melamine degradation) to AtzA (atrazine degradation) in *Pseudomonas* strains. The study identified epistatic interactions between three amino acid substitutions, highlighting a specific sequence of mutations essential for functional evolution. One substitution was a prerequisite for the two others with deviation from this mutation order resulting in loss of the parental activity and no emergence of the new activity.

Epistasis in cellular evolution (intermolecular) stems from trade-offs or constraints in the allocation of cellular resources, when increasing expression of one enzyme requires decreasing the expression of another (Cheema et al. 2017; Weisse et al. 2015). Chou et al. (2014) observed strong epistatic effects in a *Methylobacterium extorquens* strain genetically engineered to metabolise methanol. Two mutations were identified that alleviated the cost of enzyme expression, one reducing transcription and the other reducing plasmid copy number. Individually, these mutations improved the fitness of the organism but exhibited antagonistic epistasis when combined, significantly impairing overall fitness. Szappanos et al. (2016) identified an adaptation process in the ability of *E. coli* to degrade ethylene glycol involving a mutation to constitutive expression of the FucO enzyme and a subsequent tenfold amplification in copy number of a gene (*aldA*) encoding degradation of the product of FucO (glycolaldehyde). These trade-offs become evident under stress conditions necessitating resources reallocation and shifts in enzyme expression levels as identified by physical statistical analysis (Kaneko 2006; Furusawa and Kaneko 2003; Kaneko and Furusawa 2018). To restore steady-state reproduction under stress conditions, the cellular component concentration with the largest changes or highest sensitivity to the exterior stress condition will lead the evolutionary direction, driving the proportionality of changes in the concentration of other cellular components, while random mutations in other directions cancel each other out, reflecting constraints imposed by epistasis from resource reallocation and shifts in enzyme expression levels.

## Conclusions

The challenges of efficient pollutant biodegradation in microorganisms often stem from the inherent toxicity of pollutants and the absence of specialised enzymes to degrade these pollutants. However, microorganisms can adapt to pollutants by utilising pre-existing enzymes with promiscuous activities for detoxification, alone or in series. Enzymes with spacious active sites and versatility in performing various redox reactions or multiple gene copies can evolve novel activities forming metabolic pathways with opportunity for optimising gene expression to compensate for suboptimal reaction kinetics. Epistatic interactions between mutations, evident at macromolecular and cellular levels, can constrain evolutionary trajectories. Evolutionary constraints include trade-offs between the catalytic efficiency and thermal stability of enzymes and the need for proportionality among cellular components for steady-state reproduction at the cellular level. Future research should concentrate on understanding the epistatic interactions in microbial adaptation to pollutants, which can provide insight towards the direction or limitation of microbial evolution towards synthetic pollutants destruction. This insight could significantly advance the field of bioremediation, offering novel approaches for engineering microorganisms for efficient pollutant degradation.

## Data Availability

This manuscript did not generate research data.
